# Recovery Effects of Oral Administration of Glucosylceramide and Beet Extract on Skin Barrier Destruction by UVB in Hairless Mice

**DOI:** 10.3390/nu9111178

**Published:** 2017-10-27

**Authors:** Yoshihiro Tokudome, Noriomi Masutani, Shohei Uchino, Hisano Fukai

**Affiliations:** Laboratory of Dermatological Physiology, Department of Pharmaceutical Sciences, Faculty of Pharmacy and Pharmaceutical Sciences, Josai University, Saitama 350-0295, Japan

**Keywords:** beet, oral administration, glucosylceramide, skin barrier, ultra violet

## Abstract

Purified glucosylceramide from beet extract (beet GlcCer) and beet extract containing an equal amount of GlcCer were administered orally to ultra violet B (UVB)-irradiated mice, and differences in the protective effects against skin barrier dysfunction caused by UVB irradiation were compared. In the beet GlcCer group, epidermal thickening and the decrease in stratum corneum (SC) ceramide content caused by UVB irradiation were reduced. In the group that was orally administered beet extract containing glucosylceramide, effects similar to those in the beet GlcCer group were observed. Oral administration of beet GlcCer had no obvious effects against an increase in TEWL or decrease in SC water content after UVB irradiation, but there was improvement in the beet extract group. Oral administration of beet GlcCer is effective in improving skin barrier function in UVB-irradiated mice. Beet extract contains constituents other than GlcCer that are also effective in improving skin barrier function.

## 1. Introduction

The stratum corneum of skin plays a barrier function that protects organisms from external stimuli and prevents water loss. Intercellular lipids in the stratum corneum, including ceramides, cholesterol and fatty acids, are important for maintaining skin barrier function. Ceramides are the most abundant, accounting for about 40% of all intercellular lipids [1]. The number of ceramides in skin is decreased in patients with atopic dermatitis and senile xerosis, and increasing the number of ceramides is important to maintaining healthy skin [2,3]. Oral administration of plant extracts from maize [4], rice [4], konjac [5] and sugar beet [6] that contain glucosylceramide (GlcCer), a ceramide precursor, or purified GlcCer has recently been reported to be effective in improving skin barrier function not only in a hairless mouse model [5,7,8], but also in humans [5,9].

The mechanism by which administered GlcCer improves skin barrier function is not entirely clear. However, sphingoid bases, as in vivo metabolites of GlcCer, have recently been reported to activate ceramide synthesis [7,10] promoting the formation of a cornified envelope [8], and increasing tight-junction function by induction of claudin-1 [11] in human keratinocytes. With regard to species of shingoid base, rice and maize GlcCer consist primarily of 8-cis-unsaturated bonds, such as d18:2^4t^, ^8t^, d18:1^8c^ [12], while beet GlcCer consists primarily of 8-trans-unsaturated bonds, such as d18:2^4t^, ^8c^, d18:1^8t^, and t18:1^8t^ [13].

While there is no doubt about the effectiveness of GlcCer, it is not clear whether the potency of such plant extracts depends on GlcCer alone. In many cases, evaluated plant extracts have very low GlcCer contents and higher contents of other constituents. Furthermore, lipid constituents other than GlcCer present in plant extracts presumably differ considerably between plant species, plant parts (e.g., root, seeds), and method of extraction and purification. Therefore, improvement of skin barrier function by plant extracts may be related to the effects of other lipid constituents.

The present study aims to identify any differences between purified beet GlcCer and beet extract containing GlcCer and in terms of improving skin barrier function. Sugar beet is a raw material used for sugar production. The fibrous component remaining after extraction of water soluble sugar from the root is sugar beet fiber. By ethanol extraction of sugar beet fibers, beet extracts containing lipid mixtures including glucosylceramides, fatty acids and sterols can be produced as functional food materials.

This study compared differences in the effectiveness between purified GlcCer from beet extract (beet GlcCer) and beet extract containing equal amounts of GlcCer (beet extract) in preventing skin barrier dysfunction, namely, epidermal thickening, decreased stratum corneum ceramide content, increased transepidermal water loss (TEWL), and decreased stratum corneum water content, caused by UVB irradiation in hairless mice.

## 2. Materials and Methods

### 2.1. Materials

Purified glucosylceramide from beet extract (certified ≥ 99% purity) was purchased from Nagara Science Co., Ltd. (Gifu, Japan). Beet extract was provided by Nippon Beet Sugar Manufacturing Co., Ltd. (Tokyo, Japan), and was a powdered product derived from a mixture of the ethanol extract of sugar beet fiber and modified starch, containing 1.0% glucosylceramide. Beet extract also contained free sterols, sterylglycosides and free fatty acids. The analytical data on the beet extract used in this study were as follows: 0.39% schottenol, 0.37% α-spinasterol, 0.23% β-sitosterol, and 0.08% stigmastenol on HPLC; 1.3% sterylglycosides on TLC; and 0.12% palmitic acid, 0.11% linoleic acid, 0.08% oleic acid on GC (as reported by Nippon Beet Sugar Manufacturing Co., Ltd.). Ceramide NS and AS standards were purchased from Matreya LLC (Pleasant Gap, PA, USA). Ceramide NP and AP standards were obtained from Evonik Goldschmidt GmbH (Goldschmidtstrasse, Essen, Germany). Pentobarbital sodium salt was obtained from Kyoritsu Seiyaku Co. (Tokyo, Japan). Cyanoacrylate adhesives and O.C.T. compound were purchased from Daiichi Sankyo Co., Ltd. (Tokyo, Japan) and Sakura Finetek Japan Co., Ltd. (Tokyo, Japan). Phosphate-buffered saline (PBS) powder was obtained from Sigma (St. Louis, MO, USA). Silica gel 60 (Merck, Darmstadt, Germany) was used for the HPTLC plate. All other reagents were obtained commercially and used without further purification.

### 2.2. Animals

Seven-week-old male HR-1 hairless mice were purchased from Japan SLC Inc., (Hamamatsu, Shizuoka, Japan). Mice were maintained in a light- (12-h light/dark cycle) and temperature-controlled (25 ± 2 °C) barrier facility throughout the study. Mice were allowed free access to feed (Labo MR Stock; Nosan Corporation, Yokohama, Japan) and water. All animal experiments and maintenance were performed under conditions approved by the Animal Research Committee of Josai University. Mice were randomly assigned to four groups (*n* = 6, Table 1).

### 2.3. Test Sample and Schedule

Test samples were suspended in purified water containing processed starch (starch sodium octenyl succinate) as emulsifier and 0.3 mL was orally administered to each mouse using a sonde every day for 14 days. All of the glucosylceramide samples were prepared on the day of administration. 

A single dose of UVB (200 mJ/cm^2^) irradiation was applied to the back skin of each mouse using a Philips UVB lamp (TL20W/12 RS; Philips, Amsterdam, The Netherlands) at day 7 after the first administration (only one time irradiation of UVB). Collection of stratum corneum and dorsal skin sections from half of the mice was performed under anesthesia at day 3 after UVB irradiation. Table 2 shows the experimental schedule of this manuscript.

### 2.4. Measurement of Water Contents of Stratum Corneum, and TEWL

Water contents of stratum corneum and TEWL in the back skin of mice were measured daily for 14 days. Water contents of the stratum corneum were measured with a Corneometer (Courage & Khazaka, Cologne, Germany) and TEWL was assessed using a VAPO SCAN AS-VT100 RS (Asahi Techno Lab., Ltd., Yokohama, Kanagawa, Japan). Corneometer and VAPO SCAN data are given in terms of indicated arbitrary units (AU) and g/m^2^/h, respectively. 

### 2.5. Collection of Mouse Stratum Corneum

After application of anesthesia, cyanoacrylate adhesive was dropped onto slide glass, and this was adhered to the murine back skin for one minute. Slide glass was removed to collect stratum corneum sample [2].

### 2.6. Extraction Method and Lipid Analysis by HPTLC

The slide glass used to collect stratum corneum was soaked in hexane and ethanol (95:5), and was sonicated at 37 °C for 20 min. After filtering the solution, it was dried under nitrogen gas. The residue was added to 100 μL of chloroform:methanol (2:1). Briefly, stratum corneum samples were soaked in 4 mL of chloroform:methanol (2:1 *v*/*v*) and sonicated (70 W, 10 min) with a probe-type sonicator (Sonifire B-12; Branson Ultrasonics, Danbury, CT, USA). The ceramide extracted solution was dried under nitrogen gas, and was resolved in 0.4 mL of chloroform:methanol (2:1 *v*/*v*). Various ceramide extracts were separated using an HPTLC plate (Silica Gel 60; Merck, Darmstadt, Germany). HPTLC was developed twice with chloroform:methanol:acetic acid = 190:9:1 (*v*/*v*). Ceramide molecules were visualized by treatment with 10% CuSO_4_, 8% H_3_PO_4_ aqueous solution, and heating to 180 °C for 10 min. The amounts of various types of ceramide (ceramide NS, NP, AS and AP) were quantitatively determined using a densitometer.

### 2.7. Measurement of Stratum Corneum Mass

The slide glass used to collect the stratum corneum was soaked in *N*, *N*, dimethylformamide, and was sonicated for 15 min. The sonicated solution was passed through a filter that was weighed beforehand. The filter was dried for one week under vacuum. After dryness, the filter weighed again, and difference between the before and after measurements was taken as the stratum corneum mass.

### 2.8. Hematoxylin and Eosin Staining

The dorsal skin of animals was detached, cut into 0.7 cm × 0.2 cm strips with a scalpel, and embedded in OCT compound under rapid chilling on dry ice in order to prepare frozen sections. Frozen sections were cut at 0.1 μm using the Leica CM 3050 S microtome (Welzlar, Germany). Sections were subsequently stained with hematoxylin and eosin, as follows. Sections were first immersed for 30 min in a 10% formalin solution for fixation. Sections were then rinsed with water and sequentially immersed in ethanol in order to achieve dehydration. Next, sections were rinsed with water and immersed in hematoxylin solution for 10 min. Sections were again rinsed with water and immersed in an eosin solution containing acetic acid. Finally, sections were immersed in and penetrated with xylene 3 times, embedded, and observed under a microscope. Epidermal thickness was defined as the distance from the top of the stratum granulosum to the bottom of the stratum basale. Thickness of stratum corneum was measured from the top of the stratum corneum to the bottom.

### 2.9. Measurement of Epidermis Thickness

Thickness of the epidermis was measured at the horizontal midpoint of each visual field. Approximately 50 individual measurements were made along the wound margin for each histological section, and mean thickness was evaluated.

### 2.10. Data and Statistical Analysis

All results are expressed as means ± standard deviation. Statistical analysis was performed using Tukey’s post-hoc test (using SAS software version 9.2, SAS Institute, Cary, NC, USA).

## 3. Results

### 3.1. Body Weight Change after Oral Administration of Beet GlcCer or Beet Extract in UVB-Irradiated Mice

**Figure 1:**
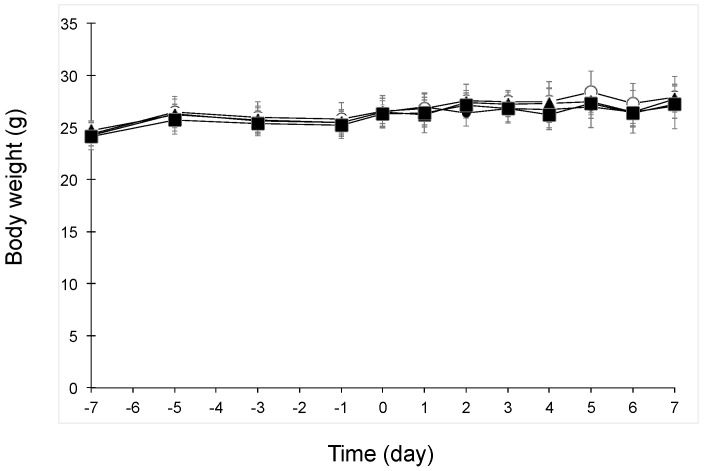
Body weight changes in UVB-irradiated mice after oral administration of beet GlcCer or beet extract. Symbols and bars represent means and standard deviation (*n* = 6 from day −7 to day 3, and *n* = 3 from day 4 to day 7), respectively. Symbols: Closed circles, Normal group (without UVB irradiation, vehicle administration); Open circles, Control group (200 mJ/cm^2^ UVB, vehicle administration); closed triangles, beet GlcCer group (200 mJ/cm^2^ UVB, beet GlcCer oral administration); closed squares, beet extract group (200 mJ/cm^2^ UVB, beet extract oral administration).

Figure 1 shows the body weight changes in UVB-irradiated mice. Body weight did not change in any groups after oral administration of beet GlcCer or beet extract.

### 3.2. Epidermal Hyperplasia Following UVB Irradiation

**Figure 2:**
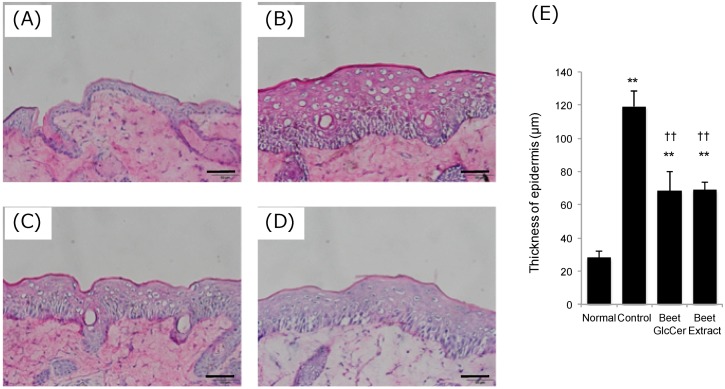
Hematoxylin and eosin staining, and thickness of epidermis in mouse skin. (**A**) Normal (without UVB irradiation, vehicle administration); (**B**) Control (200 mJ/cm^2^ UVB irradiation, vehicle administration); (**C**) Beet GlcCer (200 mJ/cm^2^ UVB irradiation, beet GlcCer oral administration); (**D**) Beet extract (200 mJ/cm^2^ UVB irradiation, beet extract oral administration); and (**E**) Epidermal thickness in UVB irradiated mice (*n* = 3). Scale bar indicates 50 μm. ** *p* < 0.01 vs. normal group, †† *p* < 0.01 vs. control group, Tukey’s post-hoc multiple comparison test.

Frozen sections of murine dorsal skin at day 3 after UVB irradiation were prepared, and hematoxylin and eosin staining of the sections was performed (Figure 2). Skin layers were thickened by UVB irradiation (normal group, 28 ± 4 μm; control group, 119 ± 9 μm; *p* < 0.01). Interestingly, beet GlcCer reduced skin thickening induced by UVB irradiation, and a similar effect was also observed in the beet extract group (beet GlcCer group, 68 ± 11 μm; beet extract group, 69 ± 5 μm; *p* < 0.01 vs. control group).

### 3.3. Comparison of Ceramide Contents in Stratum Corneum among Mouse Groups

**Figure 3:**
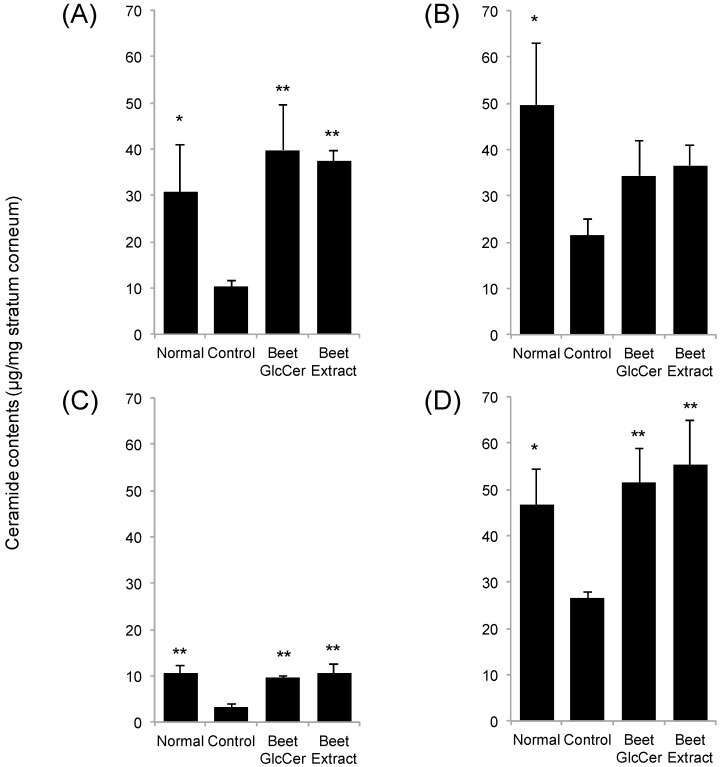
Ceramide contents in stratum corneum in UVB-irradiated (200 mJ/cm^2^) mice after oral administration of beet GlcCer or beet extract. Ceramide NS (**A**); NP (**B**); AS (**C**) and AP (**D**); respectively. Values are given as means and standard deviation (*n* = 3). * *p* < 0.05, ** *p* < 0.01 vs. control group, Tukey’s post-hoc multiple comparison test.

Quantitative analysis of ceramide contents in stratum corneum removed from the murine back skin at day 3 after UVB treatment was performed (Figure 3). UVB irradiation significantly decreased ceramide contents in stratum corneum. Remarkably, ceramide contents in UVB-irradiated mice orally given beet GlcCer were similar to those in the normal group. Similar results were observed between the beet extract group and the normal group.

### 3.4. TEWL and Water Contents in Stratum Corneum

**Figure 4:**
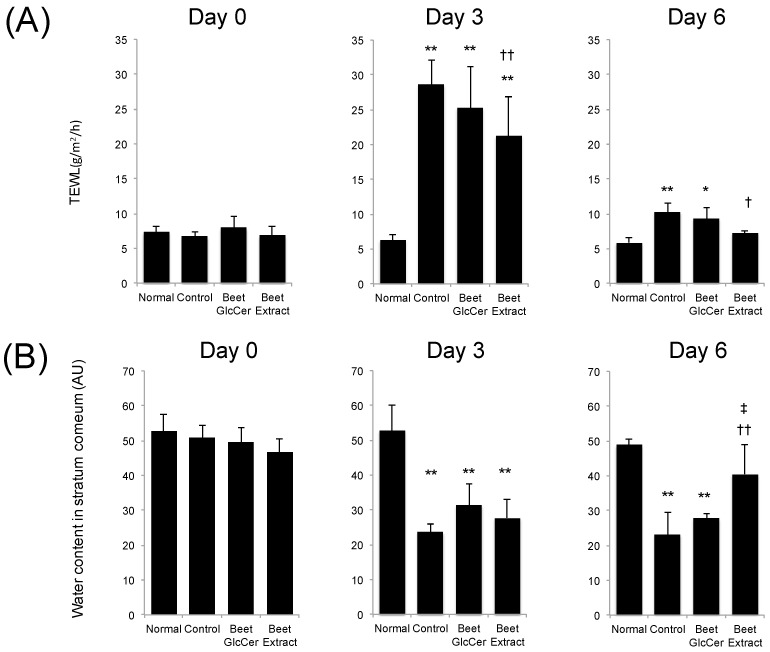
Effects of oral administration of beet GlcCer or beet extract on TEWL and stratum corneum after UVB irradiation. (**A**) Transepidermal water loss (TEWL); (**B**) water contents in stratum corneum. Values are shown as means ± standard deviation (*n* = 6 at day 0 and 3, *n* = 3 at day 6). * *p* < 0.05, ** *p* < 0.01 vs. normal group, † *p* < 0.05 vs. control group, †† *p* < 0.01 vs. control group, ‡ *p* < 0.05 vs. beet GlcCer group, Tukey’s post-hoc multiple comparison test.

Figure 4 shows the TEWL and water contents in stratum corneum at days 0, 3 and 6 after UVB irradiation in each group. TEWL in UVB-irradiated groups temporarily increased at day 3 after UVB treatment, as compared with non-UVB-irradiated mice. Mice administered beet GlcCer showed slightly lower TEWL, but the difference was not significant, when compared with the control group at day 3. Increases in TEWL in the beet extract group after UVB irradiation were significantly lower than in the control group at days 3 and 6. UVB irradiation also decreased the water content in stratum corneum in each mouse group. Although the water content in stratum corneum from the control group remained low at day 6 when compared with the normal group, early recovery of the loss of water content in the stratum corneum was observed in the beet extract group.

## 4. Discussion

This study showed that oral administration of purified GlcCer from beets was effective in preventing epidermal thickening and decreased stratum corneum ceramide content caused by UVB irradiation in hairless mice. In addition, administration of beet extract containing an equal amount of glucosyl ceramides was more effective than beet GlcCer in improving increased TEWL and decreased stratum corneum water content.

Initially, examination of skin sections on day 3 after UVB irradiation showed epidermal hyperplasia in controls. Epidermal thickness increased 4-fold when compared to the normal group. However, in the beet GlcCer group, epidermal thickness decreased to 50% when compared with controls. In the beet extract group, epidermal thickness decreased to the same degree as in the beet GlcCer group. These findings strongly suggest that inhibition of epidermal hyperplasia was due to the effects of orally administered GlcCer. Inhibition of epidermal hyperplasia by beet extract [6] and purified GlcCer from corn extract has previously been reported in a hairless mouse model of skin barrier impairment induced by an Mg-deficient HR-AD diet [7]. Our study found that beet GlcCer was also effective in preventing epidermal hyperplasia induced by UVB irradiation.

Ceramides, which account for about 40% of intercellular lipids in the stratum corneum, are important in skin barrier function [1,2,3]. A recent study found that UVB irradiation decreased skin ceramide content and increased ceramidase gene expression [14]. With oral administration of purified GlcCer from plant extracts to hairless mice fed an HR-AD diet, a 2-fold increase in epidermal ceramide synthase gene expression has also been reported [7]. Because of these previous findings, we decided to focus on stratum corneum ceramide content. In our study, we analyzed stratum corneum content of ceramides NS, NP, AS and AP, for which ceramide standards were available. The results showed a decrease in all measured ceramides at 3 days after UVB irradiation, but with oral administration of beet GlcCer, all of these decreases were reduced. Moreover, similar levels of stratum corneum ceramide content were seen in both the beet GlcCer group and the beet extract group, thus strongly suggesting that GlcCer in the beet extract contributed to these observed effects.

Haratake et al. reported epidermal cell hyperplasia followed by increased TEWL in UVB-irradiated hairless mice [15]. The increase in TEWL was reduced by DNA synthesis inhibitors, and they concluded that epidermal hyperproliferation was associated with the epidermal barrier disturbance [15]. TEWL reflects water loss from the body and is commonly used a parameter of skin barrier function. In our study, epidermal hyperplasia and increased TEWL were observed in the control group at 3 days after UVB irradiation. The effectiveness of purified GlcCer from plants in preventing TEWL has previously been reported in a hairless mouse model with barrier perturbation induced by single-dose UVB irradiation [8], in a hairless mouse model with sodium dodecyl sulfate-induced skin roughness [5], in a Mg-deficient diet-induced atopic dermatitis-like model [7], and in a tape-stripped injured skin mouse model with regard to GlcCer from konjac and maize [7]. However, there are few reports on GlcCer from beet. Haruta-Ono et al. reported on orally administered of sphingomyelin to the improvement of epidermal function in hairless mice [16]. TEWL and stratum corneum water content of sphingomyelin-administered mice showed better values than the control group. Ceramide also increased. From these results, oral application of GlcCer and sphingomyelin may have a good effect on epidermal function.

In our study, although TEWL was slightly lower in the beet GlcCer group when compared with the control group, the difference was not statistically significant. Interestingly, however, oral administration of beet extract containing an equal amount of GlcCer, when compared to the control group, significantly reduced the increase in TEWL caused by UVB irradiation. In addition, there was earlier recovery of decreased stratum corneum water content. These results strongly suggest that constituents other than GlcCer in beet extract help protect against or restore UVB irradiation-induced skin barrier dysfunction.

For example, beet-derived steryl glycosides, phytosterols and free fatty acids are present in beet extracts (described in Materials and Methods). Recent studies have reported that steryl glycosides and phytosterols inhibit UVB irradiation-induced inflammatory reactions (cytokines, matrix metalloproteinase production) in skin cells [17,18]. In UVB-irradiated skin tissue, inflammatory cytokines and chemokines such as IL-1α, TNFα and IL-8 are released from keratinocytes [19,20,21]. This is followed by activated lymphocyte infiltration into skin tissue, leading to an inflammatory reaction. Oral administration of beet extract may reduce skin barrier dysfunction after UVB irradiation more so than administration of GlcCer alone because phytosterols in beet extract prevent expansion of the inflammatory reaction in skin tissue after UVB irradiation. Confirmation of this hypothesis will require further studies.

## 5. Conclusions

Oral administration of beet GlcCer prevented epidermal hyperplasia and decreased stratum corneum ceramide content caused by UVB irradiation. In addition, our findings suggest that beet extract contains constituents other than GlcCer that are also effective in improving skin barrier function.

## Figures and Tables

**Table 1:** Experimental groups and test samples.

Group	UVB Irradiation (mJ/cm^2^)	Composition of Test Sample (mg/Mouse/Day)		
		GlcCer	Beet Ethanol Extract	Processed Starch
Normal	None	0	0	22
Control	200	0	0	22
Beet GlcCer	200	0.3	0	22
Beet Extract	200	(0.3) ^1^	8.0	22

^1^ 8.0 mg of beet ethanol extract contains 0.3 mg of beet GlcCer.

**Table 2:** Experimental schedule (d: day).

Events/Day	−7 d	−6 d	−5 d	−4 d	−3 d	−2 d	−1 d	0 d	1 d	2 d	3 d	4 d	5 d	6 d	7 d
Sample administration	◯	◯	◯	◯	◯	◯	◯	◯	◯	◯	◯	◯	◯	◯	
UV irradiation								◯							
Body weight	◯		◯		◯		◯	◯	◯	◯	◯	◯	◯	◯	◯
Water content, TEWL								◯			◯			◯	
Ceramide content											◯				
HE staining											◯				
